# Opportunities for genomic selection in American mink: A simulation study

**DOI:** 10.1371/journal.pone.0213873

**Published:** 2019-03-14

**Authors:** Karim Karimi, Mehdi Sargolzaei, Graham Stuart Plastow, Zhiquan Wang, Younes Miar

**Affiliations:** 1 Department of Animal Science and Aquaculture, Dalhousie University, Truro, Nova Scotia, Canada; 2 Department of Pathobiology, University of Guelph, Guelph, Ontario, Canada; 3 Select Sires Inc., Plain City, Ohio, United States of America; 4 Livestock Gentec, Department of Agricultural, Food and Nutritional Science, University of Alberta, Edmonton, Alberta, Canada; University of Illinois, UNITED STATES

## Abstract

Genomic selection can be considered as an effective tool for developing breeding programs in American mink. However, the genetic gains for economically important traits can be influenced by the accuracy of genomic predictions. The objective of this study was to investigate the prediction accuracies of traditional best linear unbiased prediction (BLUP), multi-step genomic BLUP (GBLUP) and single-step GBLUP (ssGBLUP) methods in American mink using simulated data with different levels of heritability, marker density, training set (TS) sizes and selection designs based on either phenotypic performance or estimated breeding values (EBVs). Under EBV selection design, the accuracy of BLUP predictions was increased by 38% and 44% for h^2^ = 0.10, 27% and 29% for h^2^ = 0.20, and 5.8% and 6% for h^2^ = 0.50 using GBLUP and ssGBLUP methods, respectively. Under phenotypic selection design, the accuracies of prediction by ssGBLUP method were 11.8% and 15.4% higher than those obtained by GBLUP for heritability of 0.10 and 0.20, respectively. However, the efficiency of ssGBLUP and GBLUP was not influenced by selection design at higher level of heritability (h^2^ = 0.50). Furthermore, higher selection intensity increased the bias of predictions in both pedigree-based and genomic evaluations. Regardless of selection design, TS sizes for GBLUP and ssGBLUP methods should be at least 3000 to achieve more accuracy than using BLUP for heritability of 0.50 and marker density of 10k and 50k. Overall, more accurate predictions were obtained using ssGBLUP method particularly for lowly heritable traits and low density of markers. Our results indicated that TS sizes should be optimized in accordance with heritability level, marker density, selection design and prediction method for genomic selection in American mink. The results provided an initial framework for designing genomic selection in mink breeding programs.

## Introduction

Availability of cost-effective and high-throughput genotyping technologies has facilitated the use of genomic selection as a useful tool to select animals for breeding in livestock species. Genomic evaluations improve the genetic gains for economically important traits through reducing generation intervals and enhancing the accuracy of selection. Furthermore, selection based on genome-wide information can be particularly effective for traits that are expensive or hard to measure and have low heritability [1]. Prediction accuracy of genomic estimated breeding values (***GEBVs***) is an important factor influencing the efficiency of genomic selection in livestock species [2]. Accuracy of prediction can be affected by several factors including extent of linkage disequilibrium [3], heritability of studied traits [4], marker density [5], effective population size [6], distribution of quantitative trait loci (***QTL***) effects [7, 8], number of animals in the training population [2] and statistical methods of prediction [9–11].

The traditional best linear unbiased prediction (***BLUP***) method is based on pedigree information and phenotypic performance of animals [12]. On the other hand, direct genomic values (***DGVs***) are predicted based on the effects of single nucleotide polymorphism (***SNP***) markers across the whole genome under the assumption of linkage disequilibrium (***LD***) between QTL alleles and SNP markers at some loci [4]. Several multi-step and single-step blending approaches have been used to combine the phenotypic and genomic information. In multi-step genomic BLUP (***GBLUP*)** methods, pseudo-phenotypes are obtained for genotyped animals using BLUP procedures at the first step, and subsequently these pseudo-phenotypes are used to solve prediction equations [13]. However, single-step GBLUP (***ssGBLUP***) blends marker-based and pedigree-based relationship matrices and makes it possible to include all genotyped and non-genotyped animals in the genetic evaluations simultaneously [14, 15].

Mink has been considered as one of the most desirable sources of pelt in the fur industry. Improvement of pelt size, fur quality, disease resistance and reproductive performance are defined as the main breeding objectives in mink production [16]. The use of modern selection methods is potentially useful to improve genetic gains and consequently economic output of mink farming. Despite the development of genomic selection programs in other domestic animals, this approach has not been developed in mink breeding programs. A simulation study using economic values of important traits in mink production indicated that genomic selection can increase the economic benefits of mink farming [17]. Recently, sequencing of the mink genome [18] has provided new opportunities for implementing genomic selection in the mink industry. Accordingly, Villumsen et al. [19] studied the accuracy of genomic predictions for fur quality traits on 2103 mink using genotyping by sequencing. However, technical aspects of designing genomic selection have not been investigated for American mink. Therefore, the main objectives of the present study were 1) to investigate the effects of different levels of heritability, marker density and selection designs on the accuracy of genomic predictions using simulated data for American mink, 2) to evaluate the accuracies of predictions using various approaches including BLUP, multi-step GBLUP and ssGBLUP in the simulated populations, and 3) to determine the optimum sizes of the training set (***TS***) for genomic selection of American mink. The results provide useful information for designing genomic selection programs in the mink industry.

## Materials and methods

### Simulation

#### Population structure

Populations were simulated using QMSim software [20] with 10k, 50k and 700k biallelic markers distributed across 14 autosomal chromosomes. At the first step, a historical population was simulated for 1000 generations with a constant size of 1000 and then the population size gradually decreased to 100 during the subsequent 50 generations to generate initial LD. This refers to typical reduction of effective population size (***Ne***) in livestock populations due to domestication [21]. The *Ne* in American mink was reported to be in the range of 17.5–70.8 [22], which was chosen as the targeted Ne in the simulation process. The same number of males and females were randomly mated at each generation in the first step. At the second step, 50 females and 50 males were randomly selected from the last historical generation to expand the size of population. The population size was enlarged during 30 generations based on random mating with an average of five kits per dam in each generation. At the final step, 260 males and 1250 females were randomly selected from the last generation of expanded population to simulate ten recent generations. Five kits per dam were simulated in each generation with a proportion of 50% male kits. Selection designs were based on either phenotypic performances or BLUP evaluations with a replacement rate of 50% for dams and 80% for sires in each generation. The chosen parameters in this step mimicked the average parameters obtained in real mink production system [16]. The breeding values were predicted using Henderson’s mixed linear model [12]. Considering the phenotypic variance of 1, traits with heritability of 0.10, 0.20 and 0.50 were simulated. Training sets including 1000, 2000, 3000, 4000 and 5000 animals were randomly selected from generations 7, 8 and 9. Small sizes of training sets were considered because the breeding programs have not been developed in the mink industry to the extent of other farm animals, and the cost of genotyping and development of SNP panels may be the major restrictions. Scenarios were defined based on different levels of heritability, marker density, selection design, TS size and prediction method. Each scenario was repeated 10 times. Pattern of LD was evaluated using r^2^ statistic. Pair-wise r^2^ was computed for all SNPs located in inter-marker distances from 0 up to 1 Mb. In addition, pedigree-based inbreeding, inbreeding based on excess homozygosity, heterozygosity levels and effective population size were calculated for each scenario using SNP1101 software [23].

#### Genome structure

The genome was consisted of 14 autosomal chromosomes with total length of 1192 cM mimicking the structure of mink genome reported by Anistoroaei et al. [24]. Phenotypes and genotypes were simulated with 5000 QTLs across the whole genome. Segregating QTLs were consisted of two, three or four alleles with MAF > 0.01 and distributed randomly across the genome. A gamma distribution (parameter shape = 0.40) was used to sample the additive genetic effects of QTLs [25]. The rates of missing marker genotypes and genotyping error were 0.05 and 0.005, respectively. Recurrent mutation rate was assumed to be 10^−5^ for both QTL and markers loci. Recurrent mutations are usually very rare between SNPs and do not contribute significantly to the reduction of LD between markers [26]. It was assumed that the genetic variance was completely explained by the additive effects of QTLs. Animal phenotypes were obtained by adding the QTL effects to a residual term sampled from a normal distribution.

### Genetic evaluations

#### BLUP

The following basic animal model was used to estimate breeding values using pedigree and phenotype information:

y=1μ+Za+e,

where ***y*** is the vector of phenotypic performances, ***μ*** is the overall mean, ***Z*** is the incidence matrix relating phenotypes to additive genetic effects, ***a*** is the vector of additive genetic effects and **e** is the vector of random residuals. It was assumed that random effects are independent and normally distributed:

$$a∼N(0,A\sigma_{a}^{2}),\;\mathrm{and}\;e∼N(0,I\sigma_{a}^{2}),$$

where ***A*** is the numerator relationship matrix, ***I*** is the identity matrix, $\sigma_{a}^{2}$ is the direct additive genetic variance, and $\sigma_{e}^{2}$ is the residual variance.

#### GBLUP

The DGVs were estimated for all genotyped individuals using the following equation:

y=1μ+Zg+e,

where ***y*** is the vector of phenotypes, ***μ*** is the overall mean, ***Z*** is the incidence matrix relating phenotypes to DGVs, ***g*** is the vector of DGV $∼N(0,G\sigma_{g}^{2})$ and ***e*** is the vector of random residuals $∼N(0,I\sigma_{e}^{2})$, where $\sigma_{g}^{2}$ is the genetic variance explained by markers, ***G*** is the genomic relationship matrix, ***I*** is the identity matrix and $\sigma_{e}^{2}$ is the residual variance. The ***G*** matrix was computed according to VanRaden method [27] as follow:

$$G=\frac{(M-P){\left(M-P\right)}^{\prime }}{2\sum_{j}^{n}p_{j}(1-p_{j})},$$

where ***M*** is the matrix of marker genotypes for each individual and ***P*** is a matrix of 2p_j_ where p_j_ is the frequency of the second allele p at locus j. The GBLUP analyses were performed using GS3 software [28].

#### ssGBLUP

The ssGBLUP method was implemented to combine information from genotyped and non-genotyped animals using the inversed of H matrix (***H***^***-1***^) blending the pedigree-based additive relationship matrix (***A*)** with genomic relationship matrix (***G*)** as follow [15, 29]:

$$H^{-1}=A^{-1}+[\begin{array}{cc} 0 & 0\\ 0 & G^{-1}-A_{22}^{-1} \end{array}],$$

where $A_{22}^{-1}$ is the sub-matrix of inversed ***A*** for genotyped individuals. Genomic relationship matrix (***G***) was built using the same approach described above for GBLUP analyses [27]. The ssGBLUP was performed using the same model implemented in BLUP analysis by replacing the ***A***^***-1***^ matrix with ***H***^***-1***^ matrix. The ssGBLUP predictions were computed based on default options in BLUPF90 software [30].

### Evaluation of predictions

Effects of all segregating markers were estimated for training sets composed of 1000, 2000, 3000, 4000 and 5000 individuals. The prediction set included 500 individuals randomly selected from the 10^th^ generation of each scenario. Accuracy of prediction was calculated as the Pearson’s correlation between true breeding values (***TBVs***) and estimated breeding values (***EBVs***) obtained from different genetic evaluation methods. The regression coefficient of TBV on EBV was used to evaluate the bias (inflation or deflation) of predictions [31]. The mean of accuracy and bias were calculated using 10 replicates of the same scenario.

## Results and discussion

### Genomic statistics

Table 1 presents the average of pedigree-based inbreeding, genomic inbreeding rates based on loss of homozygosity, observed heterozygosity and *Ne* in the recent four generations of different scenarios. Compared to phenotypic selection scenarios, reduction in heterozygosity and *Ne* were greater under EBV selection scenarios. Genomic inbreeding rates were in the range of -0.045 (±0.001) to -0.048 (±0.001) for different populations. These results were in accordance with genomic inbreeding rates (-0.150 to 0.005) reported by Thirstrup et al. [32] using 194 SNPs in 14 mink populations. In addition, the average of heterozygosities (0.343 to 0.359) in the present study was close to the observed heterozygosity (0.250 to 0.340) obtained by Thirstrup et al. [32] in feral and farm American mink.

**Table 1:** Genomic statistics (±SD) for simulated populations under phenotypic and EBV selection designs including average of pedigree-based inbreeding (F_PED_), genomic inbreeding (F_HOM_), observed heterozygosity (Ho) and effective population size (*Ne*).

		Selection design							
		Phenotypic selection				EBV selection			
Heritability	Marker density	F_PED_^1^	F_HOM_^2^	Ho^3^	*Ne*^*4*^	F_PED_	F_HOM_	Ho	*Ne*
0.10	10k	0.003±0.001	-0.046±0.002	0.355±0.015	71±9	0.035±0.002	-0.045±0.001	0.343±0.020	47±6
	50k	0.003±0.001	-0.046±0.001	0.359±0.014	72±5	0.027±0.003	-0.046±0.002	0.345±0.019	51±4
	700k	0.003±0.001	-0.048±0.001	0.358±0.013	72±4	0.023±0.002	-0.047±0.001	0.350±0.018	52±4
0.20	10k	0.003±0.001	-0.047±0.002	0.355±0.014	70±9	0.017±0.002	-0.046±0.001	0.345±0.018	53±6
	50k	0.004±0.002	-0.047±0.001	0.355±0.014	71±4	0.017±0.002	-0.046±0.001	0.348±0.018	52±5
	700k	0.004±0.001	-0.048±0.002	0.357±0.014	68±4	0.015±0.002	-0.046±0.002	0.349±0.019	54±4
0.50	10k	0.005±0.001	-0.047±0.001	0.349±0.016	61±6	0.009±0.001	-0.047±0.002	0.350±0.017	54±4
	50k	0.005±0.001	-0.046±0.002	0.350±0.015	62±6	0.010±0.002	-0.045±0.001	0.347±0.018	53±6
	700k	0.005±0.001	-0.048±0.001	0.352±0.015	64±5	0.009±0.001	-0.047±0.001	0.349±0.017	55±4
Overall		0.004±0.001	-0.047±0.001	0.354±0.003	68±4	0.018±0.008	-0.046±0.001	0.347±0.002	52±2

^1^Pedigree-based inbreeding

^2^Genomic inbreeding based on loss of homozygosity

^3^Observed heterozygosity

^4^Effective population size.

Table 2 presents the average LD of all SNP pairs for inter-marker distances from 0 to 1 Mb using bins of 0.1 Mb in EBV selection scenarios. The highest levels of LD were observed for markers distance of 0–0.1 Mb and ranged between 0.228 and 0.243. As expected, the average r^2^ decayed with increase in physical distances between markers and reached to 0.118–0.130 for 0.9–1 Mb distance. To our knowledge, genome-wide pattern of LD decay has not been reported for farmed American mink and it would be worth further investigation. However, Zalewski et al. [22] estimated the *Ne* range of 17.5–70.8 using LD method for feral American mink on the Swedish coast which was in accordance with our simulated *Ne* (47 to 72). On the other hand, lower effective sizes (7.2–34.8) were estimated for American mink populations in Spain based on LD method [33]. This discrepancy can be due to culling programs established in the Spanish regions to eradicate the invasive populations of American mink. Thirstrup et al. [32] obtained low genetic diversities, ranging between 0.250 and 0.340, using 194 SNPs in both farm and feral populations which were consistent with a low effective population size in American mink. This low *Ne* in American mink might be due to line selection in farmed mink and founder effects of farm mink escaping into feral populations [26]. Furthermore, gene flow between feral populations could be restricted due to territorial breeding [34].

**Table 2:** Average linkage disequilibrium (r^2^) for inter-marker distances from 0 up to 1 Mb^1^.

h^2^	Distance range (Mb)	Marker density					
		10k		50k		700k	
		Pairs	r^2^±SD	Pairs	r^2^±SD	Pairs	r^2^±SD
0.10	0.000–0.100	6363	0.240±0.253	12778	0.241±0.254	13126	0.243±0.261
	0.100–0.200	6391	0.210±0.235	13165	0.212±0.231	13146	0.215±0.236
	0.200–0.300	6361	0.185±0.214	13299	0.190±0.211	13053	0.191±0.212
	0.300–0.400	6249	0.170±0.201	13116	0.173±0.192	13018	0.175±0.197
	0.400–0.500	6270	0.157±0.187	13105	0.159±0.186	13037	0.162±0.183
	0.500–0.600	6225	0.152±0.177	13029	0.154±0.181	13002	0.157±0.175
	0.600–0.700	6256	0.143±0.172	13065	0.146±0.176	12977	0.148±0.173
	0.700–0.800	6229	0.134±0.162	13010	0.136±0.164	13043	0.140±0.166
	0.800–0.900	6130	0.130±0.152	13012	0.132±0.150	13003	0.135±0.157
	0.900–1.000	6174	0.123±0.151	12983	0.124±0.157	12810	0.130±0.148
0.20	0.000–0.100	6131	0.236±0.261	13273	0.238 ±0.253	13235	0.239±0.256
	0.100–0.200	6164	0.205±0.230	13204	0.206±0.231	13148	0.208±0.231
	0.200–0.300	6141	0.185±0.213	13153	0.186±0.210	13115	0.187±0.208
	0.300–0.400	6003	0.169±0.195	13036	0.171±0.190	13179	0.172±0.193
	0.400–0.500	6066	0.157±0.185	13140	0.159±0.184	12959	0.160±0.181
	0.500–0.600	6047	0.147±0.174	13091	0.149±0.175	13104	0.152±0.173
	0.600–0.700	6018	0.138±0.164	13070	0.143±0.161	13023	0.145±0.163
	0.700–0.800	6058	0.132±0.163	13038	0.134±0.160	12997	0.135±0.162
	0.800–0.900	6007	0.128±0.151	13074	0.130±0.157	12968	0.131±0.153
	0.900–1.000	6006	0.121±0.154	13033	0.122±0.148	12825	0.123±0.158
0.50	0.000–0.100	6113	0.228±0.254	13177	0.234±0.254	13187	0.235±0.261
	0.100–0.200	6012	0.199±0.223	13027	0.203±0.232	13113	0.204±0.221
	0.200–0.300	5982	0.179±0.203	13019	0.185±0.214	13076	0.185±0.203
	0.300–0.400	5976	0.168±0.185	12969	0.168±0.191	13005	0.169±0.195
	0.400–0.500	5923	0.149±0.179	12992	0.156±0.185	13107	0.157±0.183
	0.500–0.600	5972	0.146±0.167	12905	0.147±0.173	12983	0.149±0.172
	0.600–0.700	5911	0.137±0.153	12985	0.140±0.168	12919	0.144±0.163
	0.700–0.800	5910	0.129±0.152	12879	0.134±0.162	12842	0.135±0.163
	0.800–0.900	5927	0.124±0.146	12854	0.123±0.152	12745	0.130±0.158
	0.900–1.000	5877	0.118±0.149	12786	0.120±0.153	12836	0.121±0.147

^1^The average (±SD) was obtained from ten repeats of recent generation in EBV selection design scenarios.

### Accuracy of predictions

#### Selection design based on EBVs

**Fig 1:**
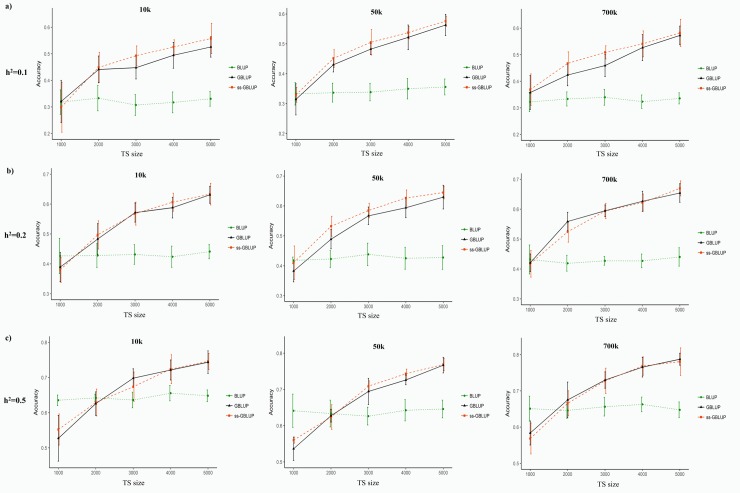
**Prediction accuracies obtained from BLUP, GBLUP and ssGBLUP methods in EBV selection design for different levels of heritability: a) h**^**2**^ **= 0.10, b) h**^**2**^ **= 0.20 and c) h**^**2**^ **= 0.50**. Accuracies were presented as a function of training set (TS) sizes for different densities of markers (10k, 50k and 700k).

Accuracy of prediction was evaluated based on the correlation between TBV and EBV. Fig 1 presents the trends of prediction accuracies as the function of TS sizes for populations which were under EBV selection in the recent generations. Prediction accuracy of BLUP method was in the range of 0.307 to 0.355 for h^2^ = 0.10, 0.417 to 0.441 for h^2^ = 0.20 and 0.620 to 0.662 for h^2^ = 0.50. Compared to BLUP, the prediction accuracy of GBLUP and ssGBLUP was increased by 38% and 44% for h^2^ = 0.10, 27% and 29% for h^2^ = 0.20, and 5.8% and 6% for h^2^ = 0.50, respectively. In accordance with our results, higher average accuracies were reported for ssGBLUP and GBLUP methods compared to traditional pedigree-based method in genomic evaluations of dairy cattle [10], pig [35], beef cattle [36, 37], broiler chicken [38] and rainbow trout aquaculture [39]. Additionally, genomic methods provided more accurate predictions than traditional BLUP using simulated data in cattle [40, 41] and pig [42].

The prediction accuracy (±SD) of GBLUP (0.357±0.067) and ssGBLUP (0.368±0.059) for h^2^ = 0.10 and marker density of 700k was higher than that was obtained from BLUP (0.322±0.036) in 1000 TS scenario (Fig 1). However, the optimum sizes of TS in genomic methods should be at least 2000 in other scenarios with low-to-moderate heritable traits (h^2^ = 0.10 and h^2^ = 0.20) to obtain more accurate predictions than BLUP. Furthermore, number of animals in TS should be at least 3000 for scenarios with high heritability of 0.50 and marker densities of 10k and 50k to achieve more accuracy than BLUP evaluation. These results indicated that TS sizes should be optimized in accordance with heritability, marker density and prediction method of genomic evaluations in American mink.

Accuracy of genomic methods was increased with enhancement in heritability levels, marker densities and TS sizes. The lowest prediction accuracy (±SD) of GBLUP (0.314±0.079) and ssGBLUP (0.298±0.084) was observed in 1000 TS scenario with 10k marker density and 0.10 heritability. On the other hand, the highest accuracy (±SD) of GBLUP (0.787±0.016) and ssGBLUP (0.780±0.038) was obtained in TS size of 5000 with h^2^ = 0.50 and marker density of 700k. Increasing effects of heritability, marker density and number of genotyped animals on the prediction accuracy has been previously confirmed in other studies on domestic animals using simulated [3] and real genomic data [43–47]. Villumsen et al. [19] used 28,336 SNP markers to evaluate the genomic merits of 639 to 752 American mink for fur quality characteristics. Considering their TS sizes, their accuracies of GBLUP predictions for body weight (h^2^ = 0.532, accuracy = 0.490), pelt length (h^2^ = 0.457, accuracy = 0.480), pelt density (h^2^ = 0.222, accuracy = 0.299) and under wool density (h^2^ = 0.163, accuracy = 0.393) were comparable with those obtained in the present study for TS size of 1000 using 10k (h^2^ = 0.20, accuracy = 0.389±0.048 and h^2^ = 0.50, accuracy = 0.527±0.065) and 50k marker density (h^2^ = 0.20, accuracy = 0.382±0.036 and h^2^ = 0.50, accuracy = 0.536±0.032). However, the predictive abilities of silky appearance of pelt (h^2^ = 0.297, accuracy = 0.820) and overall general impression (h^2^ = 0.303, accuracy = 0.685) were higher than the predicted accuracies in the present study. Furthermore, the prediction accuracies of pelt silkiness (h^2^ = 0.181, accuracy = 0.138) and pelt quality (h^2^ = 0.327, accuracy = 0.227) were lower than those obtained in our simulated data. This inconsistency can be due to the differences in the genetic architecture of these traits, number of markers, reliability of phenotypic measurements, statistical models and TS sizes in these studies.

Compared to marker density of 10k, the prediction accuracies of GBLUP and ssGBLUP were increased by 1.5% and 3.2% using 50k marker density and 6.4% and 5.7% using 700k marker density, respectively. Enhancement of marker density could improve the accuracy of genomic predictions due to increased extent of LD between markers and QTLs across the genome [3, 48]. In addition, the use of larger marker densities can reduce the sampling errors in the elements of genomic relationship matrix [6]. However, increasing marker density above 50k has provided limited improvement in the accuracy of genomic predictions in some cases e.g. dairy and beef cattle [49–52], which is in consistent with our results. Proper estimation of relationships among individuals accounting for Mendelian sampling can be the main factor of genetic improvement caused by genomic evaluation [6, 53, 54].

Overall, the prediction accuracies of ssGBLUP were 4.4%, 1.6% and 0.28% higher than those obtained using GBLUP method for heritability of 0.10, 0.20 and 0.50, respectively. These results indicated that ssGBLUP method can be more efficient particularly for lowly heritable traits and low marker density. The average reliabilities of predictions obtained from ssGBLUP were 1.8% higher than GBLUP for 16 various traits in Nordic Holstein cattle [55], which is in agreement with our results. Silva et al. [37] also obtained higher average accuracies for feed efficiency traits in Nelore cattle using ssGBLUP method (0.220 to 0.490) compared to GBLUP method (0.060 to 0.490). Furthermore, experiences with ssGBLUP in pigs indicate that ssGBLUP generally provide more accurate predictions than multi-step methods [35, 56]. In comparison with GBLUP, higher advantages were obtained for lowly heritable traits using ssGBLUP method in genomic evaluation of broiler chicken [38] and simulated pig data [57], which were in agreement with our results. More benefits of ssGBLUP can be due to simultaneous integration of both genomic and pedigree information preventing double counting of records and relationships in genomic predictions [36, 58]. Furthermore, ssGBLUP simplifies the computations and accounts for bias of preselection [58].

#### Selection design based on phenotypes

**Fig 2:**
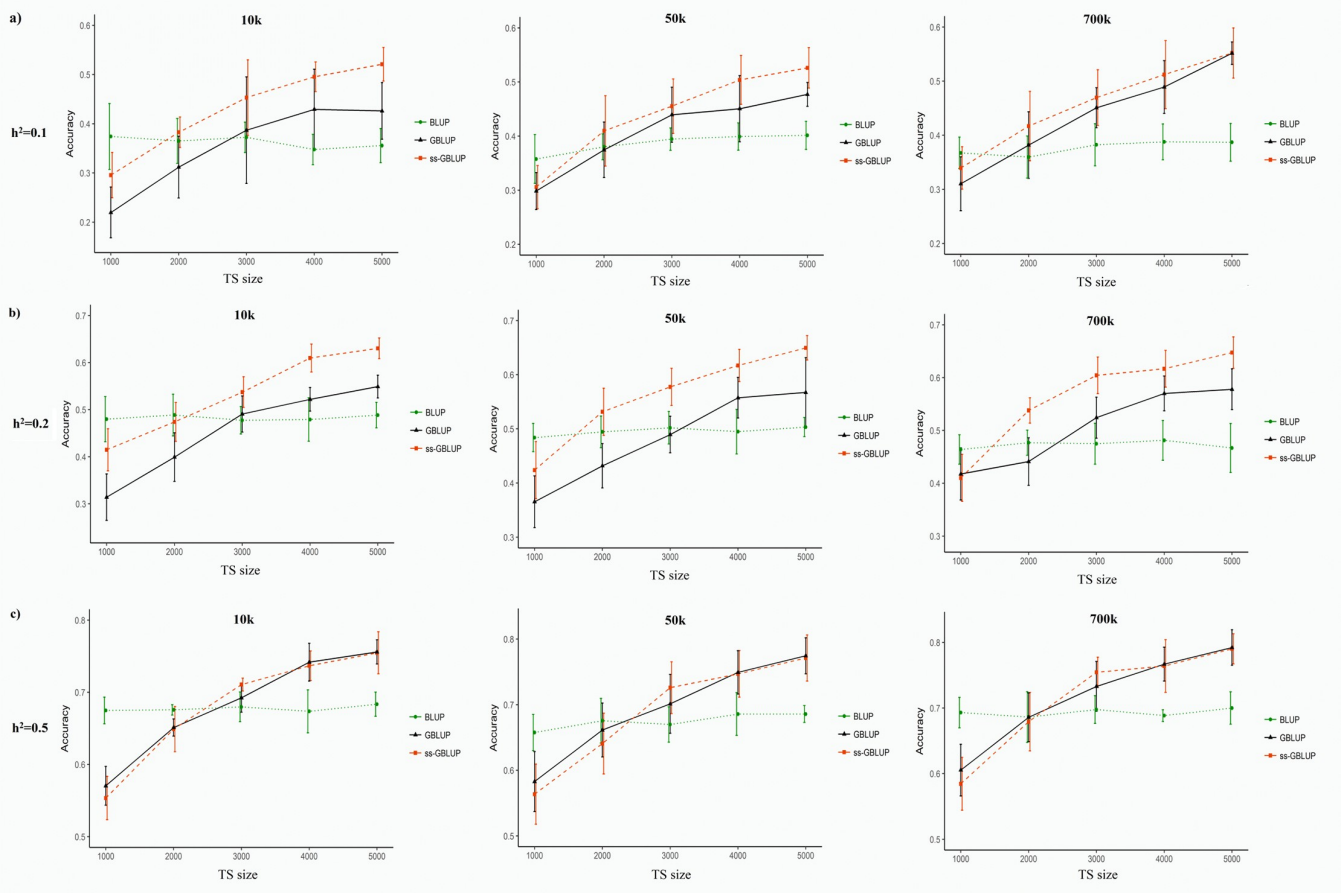
**Prediction accuracies of BLUP, GBLUP and ssGBLUP methods under phenotypic selection design for different levels of heritability: a) h**^**2**^ **= 0.10, b) h**^**2**^ **= 0.20 and c) h**^**2**^ **= 0.50**. Accuracies were presented as a function of training set (TS) sizes for different marker density (10k, 50k and 700k).

The prediction accuracies obtained from BLUP, GBLUP and ssGBLUP methods for different scenarios under phenotypic selection are presented in Fig 2. Accuracy of BLUP predictions was in the range of 0.347 to 0.401 for h^2^ = 0.10, 0.463 to 0.503 for h^2^ = 0.20 and 0.657 to 0.700 for h^2^ = 0.50. On average, the prediction accuracies of BLUP under phenotypic selection were 11.6%, 11.3% and 5.6% higher than those predicted under EBV selection design for h^2^ = 0.10, h^2^ = 0.2 and h^2^ = 0.50, respectively. For heritability of 0.50, the average accuracy of GBLUP method under phenotypic selection ranged from 0.570 to 0.792, which was 2.65% higher than those estimated under EBV selection. However, predictions of GBLUP method under phenotypic selection were 13.24% and 11.82% less accurate than those obtained under EBV selection for heritability of 0.10 and 0.20, respectively. Gowane et al. [59] reported lower accurate predictions of EBVs and GEBVs in assortative mating scenarios based on EBV (0.120 to 0.860) compared with random mating scenarios (0.210 to 0.860) in a simulation study. The lower accuracy of predictions under EBV selection designs can be induced by the reduction in genetic variation due to selection, so-called the Bulmer effect, declining the correlation between TBV and EBV in the selected individuals [60, 61]. However, GBLUP predictions were less affected by the Bulmer effect particularly at low levels of heritability due to weaker effects of selection on lowly heritable traits [59, 62].

Although TS size of GBLUP method in h^2^ = 0.10 with 10k and 50k marker densities should be >2000 to obtain more accuracy than BLUP method, the TS size of 2000 in ssGBLUP method provided higher accuracies than BLUP in the same scenarios (Fig 2). In addition, the prediction accuracies of ssGBLUP method in 2000 TS size were higher than those predicted by BLUP for heritability of 0.20 and marker density of 50k and 700k. However, the optimum size of TS for h^2^ = 0.50 should be at least 3000 to achieve more accuracy than BLUP evaluations. The results of the present study can be used as an initial framework to design and implement genomic evaluation in American mink. However, the population structure and genetic architecture of traits should also be considered to optimize the TS sizes for development of genomic evaluation system in the mink industry [63]. For example, using genomic information had no advantages on the accuracy of predictions for a trait with heritability of 0.40 in a simulated full sib population of boars within a sib-testing program [64].

Overall, the increased accuracy of predictions using ssGBLUP in comparison with GBLUP were 13.5%, 7.9% and 5.4% for marker densities of 10k, 50k and 700k, respectively. In addition, the accuracies of prediction by ssGBLUP method were 11.8% and 15.4% higher than those obtained by GBLUP method for heritability of 0.10 and 0.20, respectively. However, this difference was reduced to -0.47% for h^2^ = 0.50. These results indicated that the differences in the predictive ability of ssGBLUP and GBLUP were reduced with increased marker density and heritability. Compared to EBV selection design, the difference between the accuracy of ssGBLUP and GBLUP were increased by 7.4% and 13.45% under phenotypic selection for heritability of 0.10 and 0.20, respectively. However, the efficiency of ssGBLUP and GBLUP was not influenced by selection design for higher heritability (h^2^ = 0.50). These results revealed that ssGBLUP approach can be more efficient for improvement of lowly heritable traits such as reproduction and health traits in mink breeding programs. Therefore, considering the fact that the current selection of animals in the mink industry is mainly based on their phenotypic performances, the use of ssGBLUP method can have more advantages for mink breeding programs.

### Bias of predictions

The bias of predictions (inflation or deflation) was measured by the regression coefficient of TBV on EBV. Table 3 presents the bias of predictions obtained from BLUP, GBLUP and ssGBLUP for different levels of heritability and marker density under EBV selection design. The regression coefficient should be close to 1 to obtain more optimal prediction. As expected, the bias of predictions was improved with increase in heritability, marker density and TS sizes. On average, the regression coefficients of BLUP predictions (±SD) for heritability of 0.10 (0.695±0.028) and 0.20 (0.737±0.026) were increased to 0.779±0.058 and 0.806±0.0.059 by GBLUP, and to 0.818±0.043 and 0.841±0.047 by ssGBLUP method, respectively. However, lower differences between biases of prediction methods were observed for heritability of 0.50 (on average, 0.822±0.015, 0.853±0.051 and 0.865±0.055 for BLUP, GBLUP and ssGBLUP, respectively). On average, inflation in GBLUP and ssGBLUP methods with 10k markers were declined by 4.16% and 3.26% using 50k, and 6.62% and 3.71% using 700k marker density, respectively. In accordance with the present results, less biased predictions were reported for the single-step approach compared with multi-step GBLUP and pedigree-based methods using simulated [65] and real data [55, 62, 66]. Guarini et al. [66] observed lower biases of predictions for ssGBLUP (0.710 to 1.040) compared to multi-step GBLUP (0.630 to 1.310) for lowly heritable traits in Canadian Holstein cattle that were in consistent with our results.

**Table 3:** Regression coefficients (±SD) of true breeding values on estimated breeding values obtained from BLUP, GBLUP and ssGBLUP methods under EBV selection designs.

h^2^	Training set size	Marker density									
		10k			50k			700k			
		BLUP	GBLUP	ssGBLUP	BLUP	GBLUP	ssGBLUP	BLUP	GBLUP		ssGBLUP
0.10	1000	0.640±0.162	0.607±0.209	0.764±0.193	0.652±0.148	0.761±0.195	0.773±0.201	0.667±0.132	0.724±0.168		0.778±0.206
	2000	0.668±0.115	0.759±0.087	0.794±0.096	0.692±0.123	0.785±0.084	0.789±0.089	0.686±0.128	0.786±0.107		0.814±0.063
	3000	0.696±0.076	0.757±0.082	0.796±0.080	0.704±0.087	0.804±0.072	0.811±0.069	0.706±0.094	0.819±0.055		0.845±0.052
	4000	0.703±0.067	0.778±0.071	0.803±0.068	0.723±0.077	0.805±0.057	0.825±0.044	0.709±0.089	0.833±0.042		0.862±0.048
	5000	0.721±0.065	0.789±0.035	0.820±0.036	0.738±0.066	0.834±0.034	0.884±0.042	0.729±0.052	0.844±0.038		0.919±0.032
0.20	1000	0.707±0.105	0.702±0.124	0.749±0.103	0.718±0.114	0.725±0.117	0.782±0.116	0.733±0.098	0.748±0.116		0.805±0.093
	2000	0.716±0.097	0.770±0.070	0.788±0.091	0.696±0.071	0.798±0.067	0.818±0.086	0.704±0.066	0.836±0.097		0.826±0.084
	3000	0.722±0.068	0.840±0.068	0.818±0.066	0.731±0.059	0.822±0.066	0.843±0.076	0.728±0.063	0.838±0.045		0.887±0.063
	4000	0.758±0.066	0.846±0.055	0.854±0.058	0.763±0.054	0.843±0.040	0.880±0.057	0.750±0.051	0.875±0.038		0.890±0.052
	5000	0.789±0.046	0.872±0.037	0.893±0.029	0.767±0.042	0.883±0.033	0.893±0.039	0.774±0.046	0.901±0.029		0.898±0.045
0.50	1000	0.795±0.061	0.753±0.119	0.756±0.067	0.810±0.082	0.761±0.091	0.786±0.079	0.809±0.074	0.861±0.087		0.819±0.081
	2000	0.820±0.047	0.804±0.050	0.814±0.048	0.807±0.052	0.809±0.070	0.805±0.048	0.813±0.049	0.871±0.057		0.859±0.045
	3000	0.814±0.039	0.847±0.047	0.886±0.045	0.822±0.043	0.851±0.064	0.890±0.038	0.829±0.045	0.903±0.048		0.904±0.036
	4000	0.827±0.036	0.865±0.032	0.892±0.041	0.829±0.040	0.872±0.058	0.897±0.037	0.837±0.041	0.909±0.033		0.917±0.028
	5000	0.831±0.034	0.874±0.026	0.923±0.018	0.842±0.037	0.890±0.033	0.907±0.024	0.852±0.033	0.925±0.025	0.933±0.026	

The regression coefficients of prediction methods were higher in phenotypic selection designs compared to EBV selection designs (Table 4). In comparison with EBV selection, the bias of BLUP predictions under phenotypic selection was decreased by 25.32%, 14.84% and 4.69% for heritability of 0.10, 0.20 and 0.50, respectively. Similarly, the regression coefficients of GBLUP and ssGBLUP were increased by 16.62% and 13.67% for h^2^ = 0.10, 4.04% and 6.26% for h^2^ = 0.20, and 1.67% and 3.24% for h^2^ = 0.50, respectively. In accordance with our results, Vitezica et al. [65] obtained higher biases for genomic predictions under EBV selection (0.660 to 0.990) compared to those obtained under phenotypic selection (0.940 to 1.020) in a simulation study. In addition, Gowane et al. [59] reported higher biases of prediction in the EBV selection scenarios compared to random selection scenarios in a simulation study of accuracy and bias of genomic predictions. Ideally, very large base population of unrelated and unselected individuals is assumed in the BLUP models [12]. Under this assumption, the relationship matrix can account for the effects of selection, non-random mating and genetic drift in the population [67]. Therefore, the increased inflation of predictions in the present study could be due to violating the assumption of infinite size of base population in the simulated populations. Wrong definition of base population can increase the uncertainty of relationships and the variance of EBVs in the populations [65]. However, the bias of ssGBLUP predictions can be minimized by appropriate scaling of A^-1^ and G^-1^ and incorporating inbreeding coefficients in the genomic models [62, 65].

**Table 4:** Regression coefficients (±SD) of true breeding values on estimated breeding values obtained from BLUP, GBLUP and ssGBLUP methods under phenotypic selection designs.

h^2^	Training set size	Marker density								
		10k			50k			700k		
		BLUP	GBLUP	ssGBLUP	BLUP	GBLUP	ssGBLUP	BLUP	GBLUP	ssGBLUP
0.10	1000	0.833±0.168	0.759±0.231	0.827±0.174	0.899±0.140	0.833±0.195	0.865±0.147	0.897±0.172	0.870±0.226	1.040±0.174
	2000	0.825±0.163	0.803±0.186	0.842±0.147	0.901±0.136	0.896±0.144	0.914±0.122	0.850±0.148	1.100±0.144	1.040±0.153
	3000	0.828±0.117	0.844±0.190	0.857±0.140	0.903±0.098	0.929±0.123	0.928±0.105	0.876±0.121	1.050±0.094	1.020±0.102
	4000	0.834±0.107	0.846±0.085	0.883±0.092	0.912±0.082	0.923±0.082	0.932±0.086	0.864±0.092	0.942±0.115	0.940±0.093
	5000	0.841±0.092	0.881±0.076	0.927±0.072	0.921±0.062	0.932±0.049	0.950±0.077	0.877±0.72	1.010±0.094	0.963±0.073
0.20	1000	0.833±0.155	0.794±0.187	0.875±0.125	0.828±0.109	0.811±0.133	0.860±0.179	0.831±0.127	0.840±0.157	0.865±0.129
	2000	0.857±0.123	0.802±0.154	0.842±0.116	0.842±0.082	0.828±0.102	0.862±0.100	0.840±0.093	0.834±0.095	0.870±0.082
	3000	0.837±0.099	0.848±0.103	0.866±0.089	0.845±0.075	0.866±0.097	0.903±0.073	0.832±0.090	0.857±0.071	0.902±0.078
	4000	0.861±0.091	0.889±0.059	0.912±0.056	0.854±0.069	0.884±0.087	0.915±0.066	0.850±0.078	0.868±0.065	0.920±0.050
	5000	0.858±0.079	0.862±0.058	0.951±0.048	0.856±0.045	0.897±0.062	0.912±0.060	0.860±0.503	0.880±0.048	0.940±0.045
0.50	1000	0.864±0.062	0.775±0.060	0.803±0.054	0.809±0.065	0.785±0.079	0.806±0.058	0.886±0.096	0.873±0.098	0.857±0.068
	2000	0.863±0.049	0.817±0.057	0.814±0.033	0.818±0.057	0.840±0.059	0.820±0.047	0.873±0.077	0.907±0.067	0.877±0.070
	3000	0.866±0.034	0.842±0.049	0.885±0.027	0.829±0.054	0.843±0.040	0.890±0.041	0.894±0.070	0.904±0.039	1.010±0.053
	4000	0.873±0.029	0.864±0.046	0.896±0.023	0.840±0.045	0.880±0.038	0.893±0.047	0.887±0.058	0.933±0.042	1.010±0.051
	5000	0.884±0.023	0.897±0.038	0.903±0.016	0.833±0.041	0.911±0.032	0.919±0.025	0.895±0.060	0.935±0.031	1.030±0.051

## Conclusions

In this study, we investigated the potential advantages of genomic selection in mink breeding programs using simulated data with different levels of marker density, heritability, selection designs and TS sizes. Our results indicated that use of lower marker density (10k) can also be useful to improve genetic merit in mink farming. However, TS sizes should be optimized based on the selection designs, levels of heritability, population structure and genetic architecture of traits. In general, ssGBLUP method could provide more accurate predictions particularly for lowly heritable traits with a low density of markers. Our results indicated that higher selection intensity can increase the bias of predictions in both pedigree-based and genome-based evaluations. Overall, less biased and more accurate predictions were obtained using ssGBLUP method due to incorporating information of both genotyped and non-genotyped animals in the genetic evaluations. Genomic selection can be used as an effective strategy for improving the genetic merits of animals in mink breeding. Identification of genomic variations using sequencing technologies, development of commercial SNP panels and adaptation of statistical approaches to apply in mink breeding are the main steps to develop genomic selection programs in the mink industry. The results of this research can be helpful in designing initial frameworks of genomic selection in American mink.
